# The Antimicrobial and Wound Healing Potential of* Opuntia ficus indica L. inermis* Extracted Oil from Tunisia

**DOI:** 10.1155/2019/9148782

**Published:** 2019-04-14

**Authors:** Ikram Khémiri, Badiaa Essghaier Hédi, Najla Sadfi Zouaoui, Neyla Ben Gdara, Lotfi Bitri

**Affiliations:** ^1^Unité de Physiologie des Systèmes de Régulations et des Adaptations, Faculté des Sciences de Tunis, Université de Tunis El Manar, Tunisia; ^2^Laboratoire de Mycologie, Pathologies et Biomarqueurs, Faculté des Sciences de Tunis, Université de Tunis El Manar, Tunisia

## Abstract

**Introduction:**

* Opuntia ficus indica L. inermis* (OFI) is used in traditional medicine pharmacopeia for its richness in natural bioactive compounds. It has been proven to be effective in the improvement of the healing of laser-induced skin burns. The aim of the present study was to investigate the wound healing effect of OFI extracted oil on full-thickness skin wound.

**Materials and Methods:**

The OFI seeds were firstly isolated from mature prickly pears, washed, dried, and then cold-pressed. The antimicrobial activities of OFI seed oil were estimated* in vitro* against bacteria, yeast, and fungi. Minimum Inhibitory Concentration (MIC) and Minimal Bactericidal Concentration (MBC) were calculated. Skin wound healing was investigated using an excisional wound healing model in rats. The skin wounds of three experimental groups of rats were topically treated once/day with saline solution (control group), 0.15 mg/mm^2^ of a reference drug Esth'Elle Pharma Cicaplaie cream (reference group), and 0.6 *μ*l/mm^2^ of OFI seed oil (OFI oil group). The healing process was monitored daily and the percentage of wound contraction was calculated. A histological study was carried on skin biopsies.

**Results:**

The extracted oil has shown an interesting antimicrobial effect on* Enterobacter cloacae*, antiyeast effect against* Candida parapsilosis* and* Candida sake*, and antifungal activity against three opportunistic cutaneous molds (*Penicillium, Aspergillus, and Fusarium*). Moreover, OFI oil has shown a good wound healing effect. It prevents cutaneous infections and reduces the reepithelialization phase.

**Conclusion:**

OFI extracted oil has* in vitro* antimicrobial/fungal properties and* in vivo* wound healing activity. It seems to be efficient in the treatment of cutaneous infections and the promoting of the scarring process.

## 1. Introduction

The skin is a vital organ in the human body, since it protects it against the external environment which is rich in diverse pathogens and other factors capable of inducing diseases from the simplest to the most severe. Healing is an innate program, highly integrated into the skin that gives it the ability to regenerate after a damage caused by an injury, burn, trauma, surgery, or ulcer. It is triggered immediately in order to promptly restore the continuity and the integrity of the cutaneous barrier.

Acute wound healing is a very complex multistep process that involves commonly four overlapping and well-established succession phases; a vascular phase of haemostasis to stop the subsequent loss of blood from injured vessels, an inflammatory phenomena, a proliferation phase which includes granulation tissue formation, angiogenesis, and reepithelialization, and at last the formation of a scar that will be remodelled during the maturation of the skin architecture after wound closure [1]. In the case of a dysregulation in one or more of the steps cited above, a delay in the wound healing process or even more severe infections may occur.

Although healing is a natural phenomenon, wound management is often mandatory to prevent infections, reduce healing time, and prevent the appearance of unsightly scars, especially in the case of deep sores, bedsores, and ulcers. It stands to reason that the cutaneous and environmental microbiota may act seriously in the impairment of the wound healing [2, 3]. Tissue aseptical handling, suturing, and antiseptic therapies are sometimes insufficient to get an effective healing process. Over the recent years, clinical experiences have shown a resurgence of several infections that appeared to have been controlled and the emergence of new infectious diseases [4]. It has been reported an increase in pathogens multiresistance such as* Staphylococcus aureus *[5–7],* Streptococcus agalactiae* [8],* Enterococcus* [9],* Enterobacteriaceae* [10], and* Candida albicans* [11]. The failure of available antimicrobials to treat several infections may be due to the overprescribing of antibiotics and/or the incorrect use of them. Moreover, most of these drugs have been reported to induce harmful side effects. Therefore, it has become imperative to quest for new therapeutic agents. Phytotherapy can be considered as an alternative medicine. Several ethnopharmacological researches have contributed largely in the improvement of new medical therapies, especially in the field of cutaneous wound healing.

Plants from different genera are a natural source of a huge range of various compounds such as secondary metabolites that act as defence agents against the environmental aggressions. It is generally admitted that the beneficial effects of herbal remedies can be attributed to active constituents present either in the aerial and subterranean parts or in the whole plant, whether in crude or processed state [12].

Several active compounds like polyphenols, flavonoids, and alkaloids could be separated by liquid/liquid extraction procedures. Lipid compounds from either animal [5] or vegetal origin have been studied for their therapeutic properties [13–16]. The oils extraction procedures are diversified, including the distillation process for essential oils extraction and the mechanical pressure for fixed oils extraction. Essential oils have been widely studied and characterized for many of their properties such as antimicrobial, anti-inflammatory, and antioxidant effects [17–21].

To date, there are few studies devoted to the effective potentials of fixed oils in the wound healing process. It was reported that oils extracted from flaxseed, Pumpkin, and* Pistacia lentiscus* have beneficial therapeutic activities in the healing of skin wounds and laser-induced burns [22–25].

Prickly pear (*Opuntia ficus indica*) a plant from the* Cactaceae* family is attracting growing interest due to its richness in active compounds, precisely in the cladodes, roots, flowers, fruits, and seeds [26–30].

The purpose of the present study was to evaluate the therapeutic effects of the fixed oil, extracted by cold pressure from the seeds of* Opuntia ficus indica L. inermis* grown in Tunisia. We used an excisional wound model in rats to assess the skin healing ability of this oil. Its anti-infectious action was studied on some bacterial and fungal strains.

## 2. Materials and Methods

### 2.1. Vegetable Oil Preparation

Mature prickly pears were harvested during the month of August 2016 from the village of Zelfen in the governorate of Kasserine in central Tunisia. This delegation is known for its hard semi-arid climate conditions. The fruits were peeled manually. The seeds were isolated mechanically, washed with potable water, and dried. The oil extraction was carried out mechanically without any chemical treatment, using an oil press machine (SMIR, MUV2 65). After filtration, the first cold-pressed OFI seed oil was stored in anti-UV hermetic bottles, in order to preserve the alteration of its components.

### 2.2. *In Vitro* Antimicrobial Assays

#### 2.2.1. Antimicrobial Activity Screening

The OFI extracted oil dissolved in DMSO at 1:2 (V/V) was tested against 10 human clinical pathogenic microorganisms as listed in the following: 4 bacterial strains (*Escherichia coli, Staphylococcus aureus, Streptococcus agalactiae, *and* Enterobacter cloacae), 3 yeast strains (Candida albicans, Candida parapsilosis,* and* Candida sake), and 3 *fungi* (Aspergillus niger, Penicillium digitatum, *and* Fusarium oxysporum).* The pathogenic strains used in this work were obtained from our laboratory collection after Tunisian clinical samples isolation and identification. As positive controls we have used Ceftazidime (CAZ30) and Colicine (CL50) as antibiotics and Voriconazole (VCZ) and Amphotericin B as antifungals.

Antibacterial and antifungal tests were performed by agar well diffusion method as described [31] and broth microdilution assay using sterile Mueller–Hinton media (BioRad, France) for bacterial strains and potato dextrose agar (Bio-Rad, France) for antifungal tests. A freshly cell suspension (0.1ml) adjusted to 10^7^ CFU/ml for bacteria and 10^5^ spores/ml for fungus were inoculated onto the surface of agar plates. Thereafter, wells 6 mm in diameter were punched in the inoculated agar medium with sterile Pasteur pipettes and 50 *μ*l of the dissolved oil was added to each well. Negative controls consisted of 50 *μ*l DMSO, used to dissolve the oil. The plate was allowed to stand for 2 h to permit the oil diffusion followed by incubation at 37°C for 24 h for bacterial strains, 48 h for yeast, and 3-4 days for fungi at 28°C. The antibacterial activity was evaluated by measuring the zones of inhibition (clear zone around the well) against the test microorganisms. All tests were repeated three times.

#### 2.2.2. Determination of Minimum Inhibitory Concentration (MIC) and Minimal Bactericidal Concentration (MBC)

The minimum inhibitory concentration (MIC) of the OFI oil against pathogenic microorganisms was determined using the microdilution broth method [31]. MIC were estimated visually (absence of turbidity) and were determined with three independent measurements. Minimal bactericidal concentration (MBC) was determined from the microdilution plates used in the MIC assay, according to Celiktas et al. [18], with modifications. Aliquots (10 *μ*l) of each well without visible growth were transferred to plates containing the corresponding media culture and then incubated at 37°C for 24 h and colony growth was verified. All assays were performed in triplicate.

#### 2.2.3. Resolution of Bactericidal Activity

The antimicrobial activity of the tested oil was expressed in arbitrary units per ml (AU/ml) and it was established by an agar diffusion assay as described [32]. Briefly, a serial twofold dilution of oil in DMSO, and 50 *μ*l of each dilution were spotted onto a TSA plate seeded with about 10^5^ CFU/ml of* Staphylococcus aureus*. The AU/ml was calculated as 

AU/ml = 1000  *Χ*  D/A

  where A is the volume of the tested oil spotted on agar plate (50 *μ*l in this case); D is the reciprocal of the highest dilution showing a clear inhibition of the indicator strain.

### 2.3. Wound Healing Activity

#### 2.3.1. Animals

Healthy Wistar albino adult male rats, weighing about 170-200 g purchased from Pasteur Institute of Tunis, were used in this study. Preceding experiments, the animals were acclimated for two weeks, in the laboratory under environmentally controlled conditions; temperature 22±2°c, artificially 12 h dark/12 h light cycle, and a relative air humidity of 74 ± 2%. The rats were housed in clean plastic cages with stainless steel grid taps. They were fed with standard balanced pellet diet adapted to rats (El Badr, Utique, Tunisia) and were given potable water “*ad libitum”*. All animals were manipulated accordingly to the current guidelines for ethical control and supervision in the care and use of animals for scientific purposes and according to Medical Ethics Committee for the Care and Use of Laboratory Animals of the Pasteur Institute of Tunis, Tunisia (approval number: FST/LNFP/Pro 152012).

#### 2.3.2. Dermal Wound Healing Model

The rats were anesthetized by inhalation with diethyl ether. We used the excision wound model as described by Morton and Malone [14]. All the instruments used in the experimental surgery were autoclaved prior to the excision. Briefly, an area of 5 cm^2^ in the right subscapular region was shaved and sterilized with an ethanol solution (70% in distilled water). Full skin thickness was removed to get a wound area of approximately 225 mm^2^.

A total of 21 rats were divided into three experimental groups seven animals each.

*(i) Group1 (Control Group)*: Rats treated with normal saline solution.

*(ii) Group2 (Reference Group)*: Rats treated with 0.15 mg/mm^2^ of a reference drug “Esth'elle Pharma Cicaplaie, a healing, and anti-inflammatory cream composed of D-Panthenol, Extracts of calendula flowers, comfrey leaves, Allantoine, and titanium dioxide.

*(iii) Group 3 (OFI Oil Group)*: Rats treated with 0.6 *μ*l/mm^2^ of OFI extracted oil as described [24].

 The animals were housed individually in clean cages. The treatment was carried out once/day till the wounds of all groups were completely closed. All the treatments were topically administrated. The wound area was measured daily by placing a transparent graph paper over the wound area and tracing it. The area is then calculated by summing all squared mm (mm^2^) on the traced paper.

The wound contraction was estimated as the reduction of the original wound area size. The percentage of the wound contraction was calculated using the following formula:

(1) %  of  wound  contraction=Initial  wound  area  size–specific  day  wound  area  sizeinitial  wound  area  size×100.

The epithelialization period was estimated by the number of days required for the complete healing and the falling off of the crust from the wound area.

The evolution of the healing process was assessed macroscopically by taking digital photographs of the skin wounds, using a camera SONY DSC-W270.

#### 2.3.3. Histological Study

The cutaneous biopsies collected at the 10th day after surgery from the different experimental groups were fixed in 10% buffered formalin solution. After embedding in paraffin wax, 5 *μ*m thick sections were stained with Haematoxylin Eosin method (H&E). The slides were observed and photographed with a digital camera UCMOS 09000 KPB (Ref. FMA050) connected to an optical microscope CETI.

### 2.4. Statistical Analysis

The statistical data analysis was carried out using SPSS statistics 20.0 (SPSS Inc., Chicago, Illinois, USA). Comparisons were made using one-way analysis of variance (ANOVA).The effect of treatments was assessed by t-test. A difference was considered significant if p < 0.05. The data were expressed as mean values ± standard deviation (SD).

## 3. Results

### 3.1. Antimicrobial Effects of OFI Extracted Oil

#### 3.1.1. Antibacterial Effect

The results revealed that the tested oil was able to inhibit* Enterobacter cloacae* with a diameter of inhibition of 15.5 mm compared to 23 mm obtained by the antibacterial effect of the positive controls; Ceftazidime CAZ30 and 10 mm by Colicine CL50 used separately (Table 1). There was no antibacterial effect of OFI oil on three bacterial tested strains (*Staphylococcus aureus, Streptococcus agalactiae,* and* Escherichia coli*).

**Figure 1:**
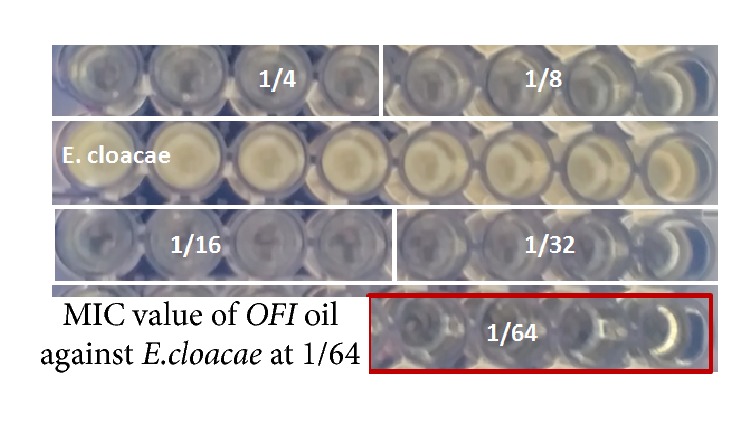
MIC value of* Opuntia ficus indica L. inermis* oil dissolved in DMSO against* Enterobacter cloacae*. MIC value of OFI oil dissolved in DMSO against* Enterobacter cloacae* was observed at a dilution value of 1/64.

In the objective to understand the bacteriostatic or bactericidal effect of the OFI oil the on bacterial strain, a volume of 10 *μ*l from the MIC well without visible bacterial growth after 24 h was transferred to TSA plates (Figure 1). The plates were incubated at 37°C for 24 h to allow bacterial growth.

The appearance of bacterial colonies on the TSA plates from the MIC well of 1/64 confirmed the bacteriostatic effect of the oil on the Gram-negative bacteria* Enterobacter cloacae. *

The bactericidal activities of the described oil, which were related to the antibacterial activities, have been reported. The results prove that the tested oil had a high bactericidal activity about 1280 AU ml^−1^. The MIC and MBC values of the oil on* Enterobacter cloacae* were, respectively, 1/64 and 1/128 (Table 2). These data suggest that the OFI oil exerts both bacteriostatic and bactericidal effects on* Enterobacter cloacae*.

#### 3.1.2. Antifungal Effects

**Figure 2:**
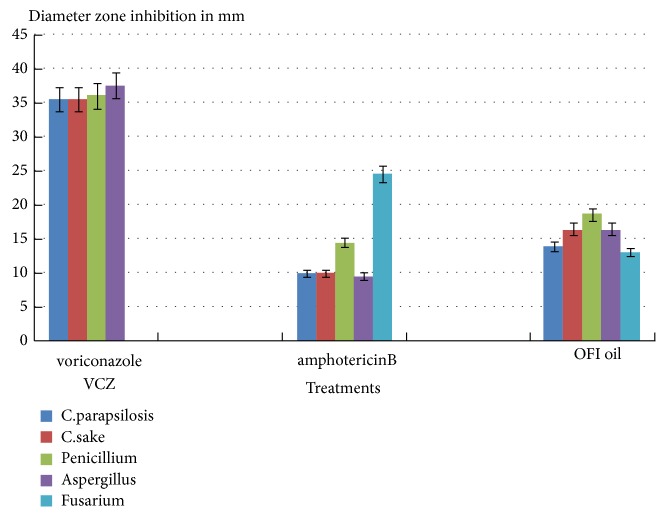
Comparison of effects of fungicidal components on the germination growth of various fungal strains used. Values measured present the diameters of the fungal growth inhibition zone (expressed in mm) using the OFI oil. Voriconazole (VCZ) and Amphotericin B were used as positive controls. OFI oil exhibited significant fungicidal effects on several fungal strains, greater than Amphotericin B (except for Fusarium) and lower than Voriconazole.

The OFI oil was able to inhibit growth of fungal pathogens. The diameter of inhibition zone of* Candida* species obtained with the OFI oil varied from 14 to 16.5 mm, respectively, against* Candida parapsilosis* and* Candida sake*, compared to 10 mm obtained with Amphotericin B and 35.5 mm with Voriconazole (VCZ) (Figure 2).

In fact, the growth of two of the three tested species of yeast (*Candida parapsilosis* and* Candida sake*) was inhibited with high MIC and MBC value, respectively, of 1/32 and 1/64 for both sensitive yeasts (Table 2).

By measuring the inhibition zone, it appears that treatment with OFI oil would be more effective against all fungal strains tested except* Fusarium* strain, compared to Amphotericin B used as a positive antifungal control. The* Fusarium* strain was more sensitive to Amphotericin B than to OFI oil.

The data have shown that VCZ was more efficient against all fungal strains with an inhibition zone diameter ranging from 35.5 mm to 37 mm. In contrast VCZ was unable to inhibit the* Fusarium* strain growth (Figure 2). These data confirmed the antifungal effect of OFI oil compared to the two antifungal positive controls.

On the other hand, the tested oil was able to reduce the growth of the three tested fungal pathogens* Aspergillus*,* Penicillium,* and* Fusarium*, with the same MIC and MBC values of 1/4 and 1/8 (Table 2).

### 3.2. Wound Healing Activity

**Figure 3:**
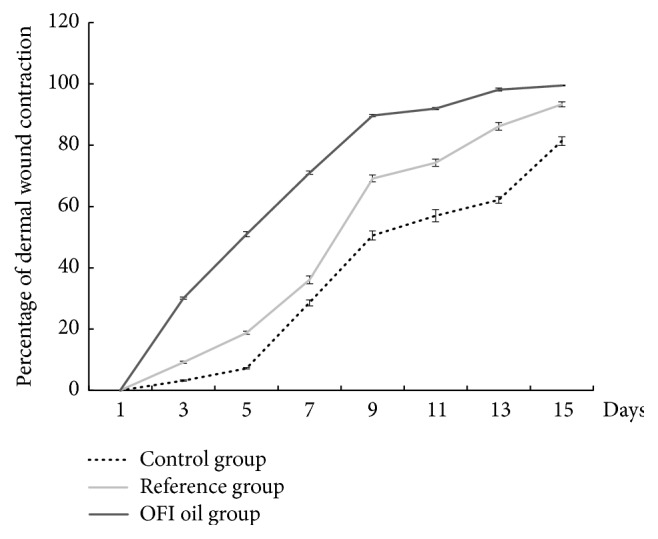
Representative graph of the percentage of the dermal wound areas contraction during the experimental process in the three studied groups of rats. OFI oil had a high curative effect (p<0.001) on skin wounds by accelerating the healing process compared to reference and control groups.

The results indicated that OFI oil has a good wound healing effect on the skin wounds. Our study has proven that OFI oil accelerated the healing process. The differences were very highly significant (p < 0.001) between the OFI oil group and the controls over the first five days after injury. We noted 50% of retraction at the 5th day after surgery with the OFI oil application versus 20% in the reference group (p < 0.001) and almost 7% in the control group (p < 0.001). A percentage of 90%, 65%, and 50% of contraction was observed at the 9th day in the three groups, respectively (Figure 3).These differences were very highly significant (p < 0.001).

**Figure 4:**
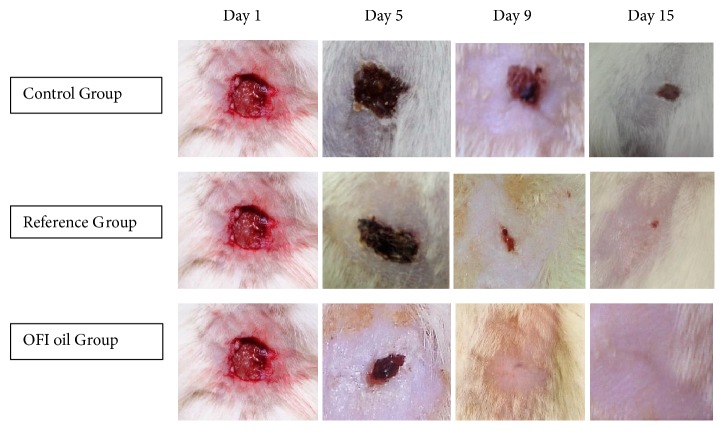
Representative photographs of macroscopic assessment of the wounds on day 1, day 5, day 9, and day 15 for the three studied groups of rats. Macroscopic assessment of the wounds showed that topical application of OFI oil resulted in a significant improvement of the healing process compared to the reference and the control groups.

The analysis of the macroscopic assessment (Figure 4) has shown a full-thickness injury induced on the 1st day. We could note that healing process was markedly delayed in the control group, in which wounds were untreated. A crust closing the wound bed has still until the last day of the experiment. Otherwise, an improvement in wound contraction rate was observed in the reference group until the 15th day after injury with complete scarring. The topical application of OFI oil during the experiment has sped up skin closure. In fact, it appeared to induce a notable gain in healing time compared to controls and the reference group, associated with an improvement of the external aspect of the scar.

### 3.3. Histology

**Figure 5:**
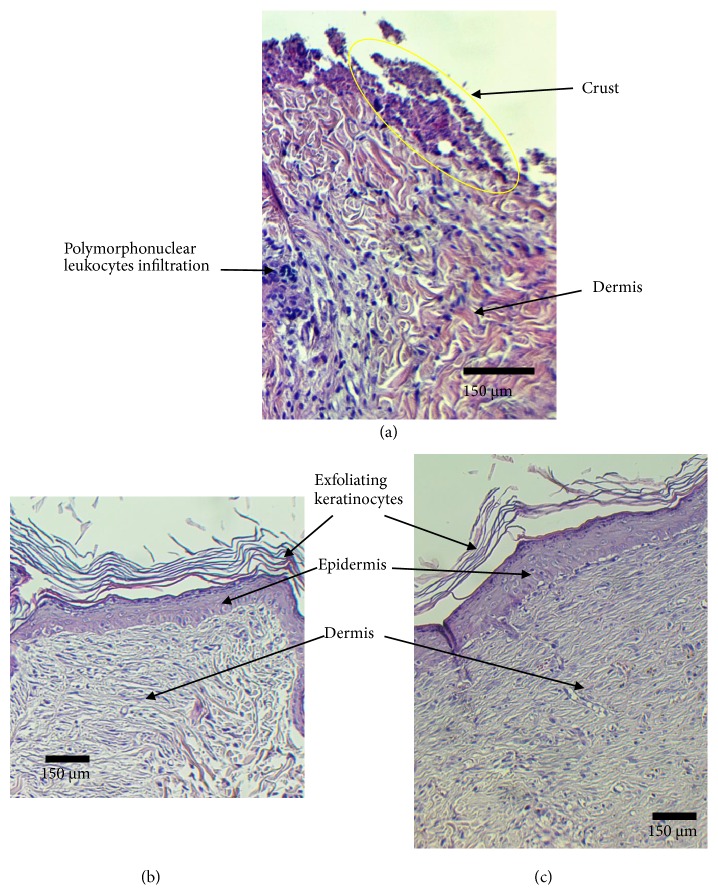
Representative images of Hematoxylin and Eosin (H&E) staining skin sections of the three experimental groups. (a) Control group; (b) reference group; (c) OFI oil group. Biopsies collected at the 10th day after surgery. OFI oil treated of the skin wounds reduced the inflammatory process and enhanced the granulation tissue regeneration and the epithelialization. The extracellular matrix components (collagen, elastin, etc.) synthesis was stimulated under this treatment compared to Control and Reference groups.

Histological analysis of the skin biopsies has shown a discontinuous epidermis in controls. We have noted the presence of an external crust with a disorganization of the dermis extracellular matrix and a notable polymorphonuclear leucocytes infiltration, confirming by the way the persistence of the inflammatory process (Figure 5(a)). In the biopsies from reference group, we could observe a continuous but thin epithelium which indicates a full reepithelialization of the skin. Nevertheless, both reference drug and OFI oil treatment led to a better dermis extracellular matrix organization compared to the normal saline treatment (Figures 5(b) and 5(c)).

## 4. Discussion

The present study was carried out in order to assess the wound healing and antimicrobial effects of OFI seed oil, using a full-thickness skin excisional model in rats. This oil can be considered an “extra-virgin seeds oil”, since the cold pressure extraction procedure does not use any chemicals and preserves all natural components. Our data have shown a spontaneous but very slow skin regeneration rate in the controls, during the first 5 days following injury. The treatment with the reference drug led to a slight improvement of the healing process, while OFI oil treatment accelerates notably the speed of wound contraction during the same period. Indeed, at the 5th day, we registered, respectively, 7%, 20%, and 50% of cutaneous wound contraction. Between the 5th and 9th day, the 3 curves have followed a parallel evolution. However, the recorded rates have shown that the oil treatment has achieved a healing rate of about 90%, while the 2nd group and the controls had only 65% and 50%, respectively, at the 9th day (Figure 3). These results could reflect that the active components of OFI oil may exert their effects especially during the early phases of healing and allow a saving in scarring time of about a week compared to controls. The high percentage of wound contraction could be related to the differentiation of myofibroblasts from fibrocytes and dermal mesenchymal multipotent stem cells (DMMSC) [33]. Indeed, it has been proven that the fibroblasts are at the origin of the generation of contraction forces and that collagen controls this process during wound healing closure [34]. These results corroborate the macroscopic observations of the evolution of healing presented in Figure 4. The scar's appearance was improved with the topical oil application. In addition, the DMMSC that are normally in a state of quiescence could be directly stimulated by the wound exudate produced during the first steps of the inflammatory phase [33]. This fluid is composed by a plethora of elements (poly-morphonuclear immune cells, lymphocytes, macrophages, platelets, debris, dead cells, proteins (albumin, globulins, etc.), growth factors, lactic acid, glucose, fibrin, wound proteolytic enzymes, collagen, elastin, and fibronectin fragments) and its extravasation in the wound bed stimulates the stem cells to express specific genes and enhances their proliferation and migration speed to the wound cavity, in order to repair the skin structure [35].

Cytoarchitectural analysis of skin biopsies from the different groups revealed the persistence of inflammatory infiltrates in the controls with a crust over the wounds. In this group, there was also a disorganization of the dermal extracellular matrix and an epidermal epithelial discontinuity, reflecting a delay in reepithelialization of the damaged skin (Figure 5(a)). The groups treated with the reference drug (Figure 5(b)) and OFI oil (Figure 5(c)) showed an advanced state of healing, confirmed by the rareness and the absence of inflammatory infiltrates, respectively, a better appearance of the extracellular dermal matrix by the resurgence of collagen and elastin fibres and myofibroblasts, parallel to the epidermis layer. In these two groups, there is a complete reepithelialization of the epidermis and the appearance of keratinocytes under three states (living, dead, and exfoliating) and the disappearance of scabs. Nevertheless, the group treated with OFI oil showed a well-regenerated epidermis by the emergence of epidermal stem cells, a basal membrane, a* stratum corneum* as well as Langerhans cells, which could be a sign of an almost complete regeneration.

These observations could be explained objectively by the OFI oil richness with unsaturated fatty acids, triacylglycerols, phytosterols, and tocopherols [26]. These active molecules could act separately or in synergy to enhance their effectiveness. Assuming that proinflammatory and inflammatory processes are prior to the tissue reconstruction phase, the active compounds present in the OFI oil, particularly *β*-sitosterol which represents 71% of total sterols (16.06 g/kg seed oil) [26], may have acted at this level. Indeed, *β*-sitosterol is renowned for several activities such as anti-inflammatory effect [36, 37]. Furthermore, this compound has been reported to have significant angiogenic potency [38] thus promoting local neovascularization in the granulation tissue, which may procure a good supply of oxygen, vitamins, minerals, antioxidants, and other active biocompounds to the wound area. Consequently, this may enhance fibroblast multiplication and so speed up the reorganization of the dermal extracellular matrix. In addition, the dense granulation tissue under the epidermis indicates an active neoangiogenesis [39].

Moreover, plant sterols have been attributed a prominent role in stabilizing the phospholipid bilayers of cell membranes, thus improving cell integrity [38].

The stimulation of the phagocytosis phenomenon by the neutrophil polynuclear leukocytes during the inflammatory process elevates oxygen consumption which contributes to the production of oxygenated free radicals such as superoxide O_2_^−^, oxygenated water H_2_O_2_, and hydroxyls OH^−^ [1]. These compounds are able to induce toxicity and damage in cell membranes and so may cause apoptosis. The high amounts of antioxidant compounds in OFI oil, such as phytosterols and tocopherols (421 mg of *γ* tocopherol per kg of seed oil [26]), may inhibit the action of the free radicals and act directly or indirectly on the production and the protection of the various actors during the phases of proliferation and regeneration of the skin (extracellular matrix, fibroblasts, Langerhans cells, keratinocytes, etc. ) [14, 39]. Moreover, the OFI oil richness with unsaturated free fatty acids (linoleic acid 60.69%, oleic acid 21.42%) and triacylglycerols, mainly LLL and OLL [26], may protect against the peroxidation and could assure the reconstruction of the phospholipids of the cell walls. At this regard it has been reported that linoleic acid application was able to inhibit lipid peroxidation, thus reducing the delay effect on damaged-tissue repair [40]. Furthermore, it has been described that linoleic acid is the precursor of arachidonic acid which is the major polyunsaturated fatty acid in the skin. Arachidonic acid has been cited as the precursor of biologically active inflammatory mediators (prostaglandins, thromboxane, and leukotrienes) which may stimulate angiogenesis, fibroplasia, and extracellular matrix remodelling [23, 35].

It is well known that a high level of hydration is required during the wound healing process. It has been proven that fatty acids and triacylglycerols enhance skin hydration level by reducing the trans-epidermal water loss [28]. The topical application of OFI oil combined with hyaluronic acid normally present on the sides of the wound cavity [1] may protect the cells against the dehydration and promote the scarring process, especially by promoting the advancement of the epithelium over the granulation tissue.

It is obvious that wounded skin remains vulnerable to invasive microbiota. Actually, it has been described that the exudates could be contaminated by the cutaneous bacteria, yeast, and fungi, especially during chronic wounds, and, in consequence, the healing process may be slowed or complicated by infections [1]. Overall, OFI tested oil exhibited significant antimicrobial effects against bacteria (*Enterobacter cloacae*), yeasts (*Candida parapsilosis* and* Candida sake*), and fungi (*Aspergillus niger*,* Penicillium digitatum,* and* Fusarium oxysporum*). These results may confirm the protective effect of OFI tested oil against infection. Nevertheless, the mechanisms of action of this oil against the pathogens should be more elaborated and comprehended. For antibacterial agents it would be preferable to exhibit bactericidal action rather than a bacteriostatic way. However, this does not exclude that these antimicrobials may exert their effects through both mechanisms of action [41]. The* in vitro* microbiological determination of bactericidal and bacteriostatic actions may be influenced by various factors such as bacterial specie or bacterial density, growth conditions, and test duration. In this work, on the basis of the MIC value determination and the agar diffusion method, we have demonstrated both bacteriostatic and bactericidal actions of the OFI oil against the tested clinical pathogenic bacteria. As cited above, OFI oil is rich in fatty acids [26], in particular linoleic and oleic acids. Since 1972, bactericidal and antifungal activities of free fatty acids have been proved [42]. Dilika et al. [43] have reported a significant antibacterial activity of linoleic and oleic acids isolated from* Helichrysum pedunculatum*, especially against gram-positive bacteria. Furthermore, it seems that the free fatty acids may act on one hand by inhibiting membrane enzymatic activities such as glucosyltransferase and on the other hand by activating autolytic enzymes in the pathogen cell wall. This may lead to a disorganization of the membrane phospholipids bilayer, which may result in a notable decrease in nutrient uptake, the inhibition of bacterial cell growth (bacteriostatic action), and an increase in membrane permeability and cell lysis (bactericidal action) [44]. Moreover, the free fatty acids seem to be able to induce uncoupling oxidative phosphorylation and a disruption of electron transport chain through the pathways involved in ATP production and regeneration [44, 45]. Moreover, the antimicrobial efficiency of OFI oil could be explained by its richness in phytosterols and especially in beta-sitosterol [26]. It has been described that this sterol could inhibit the growth of certain microorganisms, probably by interfering with the cell membrane sterols, and so altering its permeability to nutrients, which may result in the disruption of the cell vital pathways and so induce necrosis of pathogenic cells [38, 46].

Even if all therapeutic claims attributed to OFI oil are supported by bibliographic data, it remains that future investigations are required to better assess the effects of the active compounds in this oil, in particular during the first phases of the wound healing and the mechanisms by which they could modulate the function of the different actors involved in the healing process.

## 5. Conclusion

The results of the current study have shown the effectiveness of OFI extracted oil in the improvement of cutaneous wound healing of a full-thickness injury, by enhancing the speed of wound contraction, the complete reepithelialization, and improving the external scar's aspect. The antimicrobial effect of this oil could prevent wound area infections, thus preventing a delay in the healing process.

## Figures and Tables

**Table 1:** Diameters of the bacterial growth inhibition zone using the OFI oil. OFI oil showed a microbial growth inhibitory effect against *Enterobacter cloacae*, greater than Colicine and lower than that of Ceftazidime.

Bacterial strain	Ceftazidime (CAZ 30)^*∗*^ (mm)	Colicine (CL 50)^*∗*^ (mm)	OFI oil (mm)

*Enterobacter cloacae*	23 ± 1.41	10 ± 0	15.5 ± 0.35

^**∗**^(CAZ30) and (CL50) were used as positive controls.

**Table 2:** Minimum inhibitory concentration (MIC) and minimum bactericidal concentration (MBC) of the OFI seed oil against microorganisms. The tested oil had antibacterial activities against *Enterobacter cloacae*, anti-yeast effects against *Candida parapsilosis* and *Candida sake*, and antifungal activity by reducing growth of three fungal pathogens *Aspergillus*, *Penicillium,* and *Fusarium*.

Microorganisms	Presence of activity	MIC	MBC
*Bacteria *			
* Escherichia coli *	-		
* Streptococcus agalactiae *	-		
* Staphylococcus aureus*	-		
* Enterobacter cloacae*	+	1/64	1/128
*Yeasts*			
* Candida albicans*	-		
* Candida parapsilosis*	+	1/32	1/64
* Candida sake*	+	1/32	1/64
*Fungi*			
* Aspergillus niger*	+	1/4	1/8
* Penicillium digitatum*	+	1/4	1/8
* Fusarium oxysporum*	+	1/4	1/8

(+): presence of activity; (-): absence of activity.

## Data Availability

The data used to support the findings of this study are available from the corresponding author upon request.
